# Epidemiological and clinical considerations on *Clostridium difficile* colitis in Braşov County

**DOI:** 10.1186/1471-2334-14-S7-O26

**Published:** 2014-10-15

**Authors:** Maria Elena Cocuz, Ligia Rodina, Iuliu Gabriel Cocuz

**Affiliations:** 1 Faculty of Medicine, Transilvania University, Braşov, Romania; 2 Infectious Diseases Hospital, Braşov, Romania; 3 University of Medicine and Pharmacy Tîrgu Mureş, Romania

## Background

*Clostridium difficile* is currently the most frequent cause of nosocomial diarrhea but also cause of disease in the community, antibiotic therapy and hospitalizations related; advanced age (over 60 years), malignancies, chronic kidney disease are also risk factors for infection with *Clostridium difficile*. The incidence of *Clostridium difficile* increases all over the world. The complications of the disease can be severe (fulminant colitis, toxic mega colon, colonic perforation, sepsis), with need for admission to intensive care unit and risk of death. The aim of this study was to analyze some epidemiological and clinical aspects on colitis with *Clostridium difficile* in patients admitted into the Infectious Diseases Hospital of Braşov.

## Methods

It is a retrospective study, on 106 cases with *Clostridium difficile* infection, admitted in the Infectious Diseases Hospital of Braşov during November 2012 – April 2014. We analyzed: age of patients, previous hospitalizations, recent use of antibiotics, clinical forms of the disease, laboratory disturbances, frequency of relapses and deaths.

## Results

The age of the patients ranged between 18 and over 91 years old, 72.64% were over 60 years old and 16.98% over 80. 78.30% of the patients had had previous recent hospitalization in medical or surgical units. A percentage of 81.13% of patients reported previous use of antibiotics (in hospital but also in community, especially fluoroquinolones and cephalosporins). We found severe clinical forms in 20.75% of cases; relapses were shown in 16.98% of the patients and the frequency of deaths was 4.72%. The most important laboratory disturbances were: leukocytosis in 53,92% cases (14.71% over 20,000 WBC/cmm), high level of serum creatinine in 41.41% patients (over 3mg% in 7.07% patients) and hypoproteinemia in 77.27% cases (11.36% cases under 4.5 mg%).

## Conclusion

1. Patients admitted with *Clostridium difficile* colitis were elderly in most cases, requiring more complex care tailored to specific age and age-related pathology.

2. Increased frequency of hospitalizations and use of antibiotics were observed in the recent medical history of the patients.

3. Severe forms of the disease and relapses were frequent, a fact that led to an increased hospitalization period.

4. A documented and argued choice of the antibiotics therapy for different diseases is necessary in patients who have risk factors for the occurrence of *Clostridium difficile* colitis, avoiding the frequent use of fluoroquinolones and cephalosporins.

